# Persistent Interleukin‐1β Elevation in Post‐COVID‐19 Patients: Findings From a Nationwide Registry Study in Japan

**DOI:** 10.1002/npr2.70082

**Published:** 2026-02-27

**Authors:** Naoki Takamatsu, Hiroki Kimura, Mari S. Oba, Hiroyuki Chiba, Ikue Umemoto, Yuki Moriyama, Nobuaki Matsunaga, Shinichiro Morioka, Daisuke Mori, Kazuhiro Hara, Aya Ogura, Kazufumi Yoshida, Hirohisa Watanabe, Satoshi Maesawa, Masashi Ikeda, Masahisa Katsuno, Norio Ohmagari, Masaki Takao, Shinsuke Kito, Norio Ozaki, Hironori Kuga

**Affiliations:** ^1^ National Center for Cognitive Behavior Therapy and Research National Center of Neurology and Psychiatry Kodaira Tokyo Japan; ^2^ Department of Psychiatry Nagoya University Graduate School of Medicine Nagoya Aichi Japan; ^3^ Department of Clinical Data Science, Clinical Research & Education Promotion Division National Center of Neurology and Psychiatry Kodaira Tokyo Japan; ^4^ Disease Control and Prevention Center National Center for Global Health and Medicine Tokyo Japan; ^5^ AMR Clinical Reference Center National Center for Global Health and Medicine Tokyo Japan; ^6^ Brain and Mind Research Center Nagoya University Nagoya Aichi Japan; ^7^ Department of Neurology, Japanese Red Cross Aichi Medical Center Nagoya Daini Hospital Nagoya Aichi Japan; ^8^ Department of Neurology Nagoya University Graduate School of Medicine Nagoya Aichi Japan; ^9^ Department of Neurology Fujita Health University Hospital Toyoake Aichi Japan; ^10^ Department of Neurosurgery National Hospital Organization, Nagoya Medical Center Nagoya Aichi Japan; ^11^ Department of Clinical Laboratory and Internal Medicine National Center of Neurology and Psychiatry Kodaira Tokyo Japan; ^12^ Department of Psychiatry National Center of Neurology and Psychiatry Kodaira Tokyo Japan; ^13^ Department of Clinical Psychology National Center of Neurology and Psychiatry Kodaira Tokyo Japan; ^14^ Pathophysiology of Mental Disorders Nagoya University Graduate School of Medicine Nagoya Aichi Japan; ^15^ Institute for Glyco‐core Research (iGCORE) Nagoya University Nagoya Aichi Japan

**Keywords:** biomarkers, COVID‐19, inflammation, interleukin‐1beta, SARS‐CoV‐2

## Abstract

**Aim:**

COVID‐19 has been associated with dysregulated immune responses, with increasing evidence indicating sustained inflammasome activation and subsequent pro‐inflammatory cytokine production. This study aimed to characterize the temporal profile of inflammatory markers, particularly interleukin (IL)‐1β, in post‐COVID‐19 patients compared with pre‐pandemic healthy controls, using data from the Psychiatric Symptoms for COVID‐19 Registry Japan (PSCORE‐J).

**Methods:**

Blood samples were analyzed from 119 post‐COVID‐19 patients (median age 45 years) recruited during 2023 and 374 pre‐pandemic healthy controls (median age 65 years). For post‐COVID‐19 patients, samples were collected at baseline, 3 months, and 9 months. Multiple inflammatory markers were assessed, including IL‐1β, IL‐6, IL‐8, IL‐10, IL‐12, TNF‐α, IFN‐γ, IFN‐β, IP‐10, ACE2, and eotaxin. Age‐ and sex‐adjusted analyses were performed on log‐transformed IL‐1β levels.

**Results:**

IL‐1β levels were significantly elevated in post‐COVID‐19 patients compared with healthy controls across all age groups (under 30s: 0.69 ± 0.33 vs. 0.25 ± 0.03; 30s: 0.70 ± 0.63 vs. 0.26 ± 0.09; 40s: 0.84 ± 0.76 vs. 0.30 ± 0.23; 50s: 0.67 ± 0.65 vs. 0.26 ± 0.10; 60 or over: 0.54 ± 0.30 vs. 0.26 ± 0.23 pg/mL). This elevation was sustained throughout the 9‐month follow‐up (baseline: 0.500 [0.33–0.890]; 3 months: 0.630 [0.28–1.290]; 9 months: 0.54 [0.29–0.96] pg/mL) compared with controls (0.24 [0.21–0.27] pg/mL). Other inflammatory markers showed either no significant differences or were paradoxically lower in patients.

**Conclusion:**

SARS‐CoV‐2 infection is associated with persistent elevation of IL‐1β levels that remains stable over a 9‐month period, suggesting sustained inflammasome activation. These findings provide novel insight into post‐COVID‐19 inflammatory processes and may have important implications for understanding both acute and chronic manifestations of the disease.

**Trial Registration:**

Japan Registry of Clinical Trials: jRCT1030220711

## Abstract 2

Post‐COVID‐19 patients show persistent elevation of interleukin‐1β compared to healthy controls, sustained over 9 months without normalization. This low‐grade inflammatory response suggests ongoing immune activation following SARS‐CoV‐2 infection, providing insights into potential mechanisms underlying post‐COVID‐19 neuropsychiatric symptoms and highlighting the need for longitudinal inflammatory monitoring.

## Graphical abstract

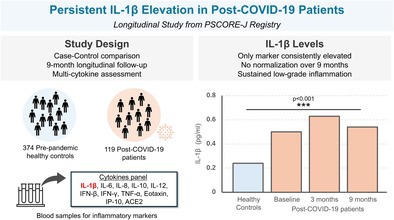

## 1 Introduction

Post‐COVID‐19 condition (PCC), as defined by the World Health Organization [1], represents a significant global health challenge. Neuropsychiatric symptoms are among its most prevalent features [2]. A 3‐year follow‐up study has highlighted their chronic nature and significant burden in disability‐adjusted life years (DALYs), with neurological manifestations accounting for 32.2 per 1000 cases, even among nonhospitalized patients [3].

Viral infections typically trigger immune responses characterized by increased production of pro‐inflammatory cytokines. In COVID‐19, this response can become dysregulated, as SARS‐CoV‐2 may evade initial type I interferon (IFN) responses through mechanisms including mitochondrial dysfunction and altered calcium signaling [4]. Notably, SARS‐CoV‐2 has been associated with engagement of inflammasome pathways and increased IL‐1β/IL‐18 signaling in some contexts [5, 6], but whether long‐term NLRP3 activation persists in post‐COVID populations remains uncertain. We therefore focused on IL‐1β as a sensitive readout of inflammatory tone while avoiding mechanistic over‐attribution.

To address these gaps, the Psychiatric Symptoms for COVID‐19 Registry Japan (PSCORE‐J) [7] was established as a systematic nationwide initiative with three fundamental aims: building a clinical database of post‐COVID‐19 cases, investigating pathophysiological mechanisms, and developing therapeutic strategies. This study reports inflammatory marker profiles from PSCORE‐J, focusing on IL‐1β, which showed the most significant alterations.

## 2 Methods

This study analyzed blood samples from two independent cohorts. Samples from post‐COVID‐19 patients were prospectively collected through PSCORE‐J with longitudinal sampling from January to December 2023. In contrast, samples from healthy controls (HC) were retrospectively obtained from a pre‐pandemic cohort recruited in 2020 at the Brain and Mind Research Center of Nagoya University [8]. For post‐COVID‐19 patients, blood samples were collected at three time points: baseline, 3 months, and 9 months postinfection. All samples were analyzed for multiple inflammatory markers including angiotensin‐converting enzyme 2 (ACE2), IFN‐γ, interleukin (IL)‐10, IL‐1β, IL‐6, IL‐8, tumor necrosis factor (TNF)‐α, eotaxin, IL‐12, interferon γ‐induced protein 10 (IP‐10), and IFN‐β. Age‐ and sex‐adjusted analyses were performed using analysis of covariance (ANCOVA). Bonferroni correction was applied for age‐stratified IL‐1β comparisons.

## 3 Results

The study included data from 119 post‐COVID‐19 patients (median [interquartile range (IQR)]: 45 [35–52] years, 51 males, 68 females) and 374 HC (median [IQR]: 65 [52–72] years, 131 males, 243 females). For post‐COVID‐19 patients, the median [IQR] time from the most recent infection to baseline sampling was 185 [139–334] days (mean (standard deviation, SD): 249 (184) days). Among those with available data, 66 (82.5%) were vaccinated and 48 (60.0%) were married. Overall clinical severity assessed by the Clinical Global Impression‐Severity scale (CGI‐S) was distributed as follows: 38 (56.7%) minimal to mild symptoms (CGI‐S 1–3), 29 (43.3%) moderate‐to‐severe symptoms (CGI‐S 4–7). A total of 236 samples were collected from post‐COVID‐19 patients across all time points.

**FIGURE 1:**
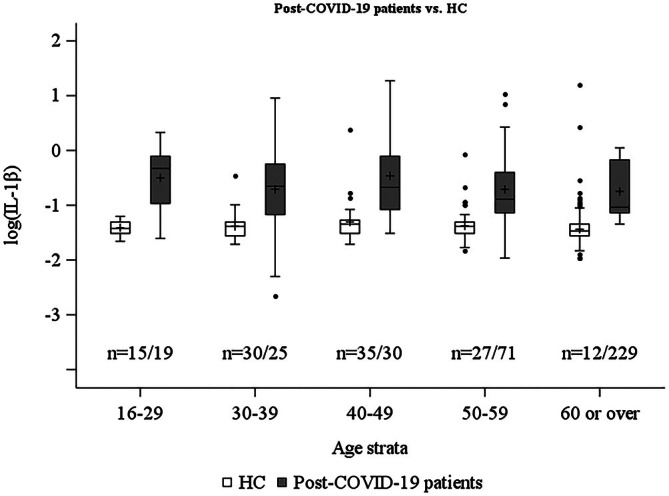
Age‐stratified log (IL‐1β) levels in post‐COVID‐19 patients and healthy controls. Box plots showing log‐transformed IL‐1β levels across age groups in post‐COVID‐19 patients (light gray, *n* = 119) and healthy controls (dark gray, *n* = 374). Boxes represent the interquartile range (25th–75th percentiles) with a median line; whiskers extend to the most extreme values within 1.5 times the interquartile range; dots beyond whiskers represent outliers. Crosses (+) indicate mean values, *p* < 0.01 for all age group comparisons (age‐ and sex‐adjusted ANCOVA with Bonferroni correction). HC, healthy controls.

The mean (SD) values of IL‐1β by age group for post‐COVID‐19 patients vs. HC, respectively, were as follows: under 30s, 0.69 (0.33) vs. 0.25 (0.03); 30s, 0.70 (0.63) vs. 0.26 (0.09); 40s, 0.84 (0.76) vs. 0.30 (0.23); 50s, 0.67 (0.65) vs. 0.26 (0.10); and 60 or over, 0.54 (0.30) vs. 0.26 (0.23) pg/mL. Post‐COVID‐19 patients showed significantly higher log‐transformed IL‐1β levels across all age groups compared with HC (all *p* < 0.01, age‐ and sex‐adjusted ANCOVA) (Figure 1).

**FIGURE 2:**
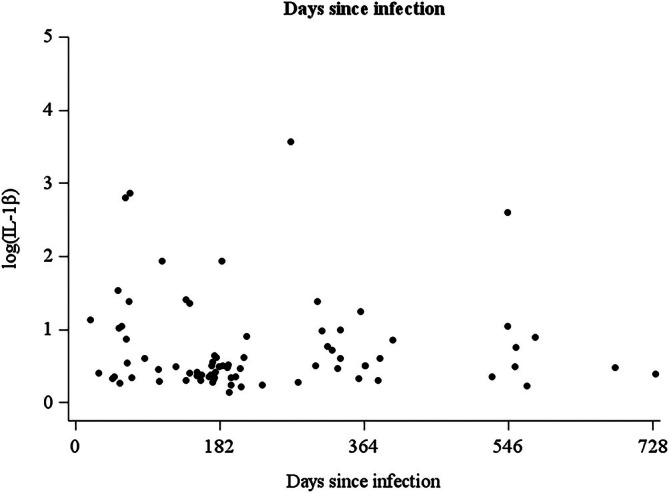
Relationship between postinfection duration and IL‐1β levels at baseline. Scatter plot showing IL‐1β levels (pg/mL) plotted against days since SARS‐CoV‐2 infection for post‐COVID‐19 patients at baseline (*n* = 119). The dashed horizontal line indicates the median IL‐1β level for the patient cohort. IL‐1β values show higher levels and greater variability in patients with shorter postinfection duration, with a tendency toward convergence over time.

To examine the relationship between postinfection duration and IL‐1β levels at baseline, we plotted individual IL‐1β values against days since infection (Figure 2). IL‐1β values showed higher levels and greater variability in patients with shorter time since infection, with a tendency toward convergence over time, although this relationship was neither linear nor homoscedastic.

For longitudinal analysis, the number of patients decreased at each follow‐up interval (baseline: *n* = 119; 3 months: *n* = 79; 9 months: *n* = 38). Compared with IL‐1β levels in HC (median [IQR]: 0.24 [0.21–0.27] pg/mL), levels in post‐COVID‐19 patients remained consistently elevated throughout the study period (baseline, 0.50 [0.33–0.89]; 3 months, 0.63 [0.28–1.29]; 9 months, 0.54 [0.29–0.96] pg/mL), without demonstrating a trend toward normalization over the 9 months.

Multiple inflammatory markers were assessed in both cohorts (Table 1). Among all measured markers, four showed statistically significant differences between post‐COVID‐19 patients at baseline and HC: IL‐1β (*p* < 0.001), IL‐6 (*p* = 0.013), IL‐10 (*p* = 0.003), and IFN‐γ (*p* = 0.045). Notably, IL‐1β was the only marker elevated in post‐COVID‐19 patients, while IL‐6, IL‐10, and IFN‐γ were paradoxically lower than HC. Other inflammatory markers showed no significant differences between groups.

**TABLE 1:** Inflammatory marker levels in post‐COVID‐19 patients and healthy controls.

	Healthy controls	Post‐COVID‐19 patients			*p*
	(*n* = 374)	Baseline	3 months	9 months	
		(*n* = 119)	(*n* = 79)	(*n* = 38)	
IL‐1β (pg/mL)	0.24 [0.21–0.27]	0.50 [0.33–0.89]	0.63 [0.28–1.29]	0.54 [0.29–0.96]	< 0.001
IL‐6 (pg/mL)	0.74 [0.47–1.11]	0.67 [0.36–1.12]	0.54 [0.28–0.99]	0.46 [0.23–0.87]	0.013
IL‐8 (pg/mL)	4.16 [3.37–5.18]	3.03 [2.11–4.35]	3.22 [2.04–4.33]	2.58 [1.77–4.03]	0.480
IL‐10 (pg/mL)	0.52 [0.45–0.61]	0.47 [0.42–0.55]	0.44 [0.38–0.53]	0.48 [0.43–0.52]	0.003
IL‐12 (pg/mL)	149.1 [111.7–200.9]	—	—	—	—
IFN‐β (pg/mL)	32.1 [16.4–62.7]	31.6 [12.2–73.5]	42.5 [22.0–70.8]	31.6 [15.0–50.8]	0.198
IFN‐γ (pg/mL)	12.7 [9.3–19.0]	10.2 [7.9–15.4]	7.0 [4.2–10.7]	6.2 [4.3–10.2]	0.045
TNF‐α (pg/mL)	0.57 [0.45–0.71]	0.53 [0.41–0.72]	0.43 [0.34–0.59]	0.45 [0.37–0.54]	0.484
Eotaxin (pg/mL)	354.3 [297.2–443.3]	356.3 [286.8–453.3]	345.6 [309.1–442.9]	370.1 [306.6–418.9]	0.807
IP‐10 (pg/mL)	514.7 [387.2–666.9]	430.7 [351.9–580.5]	387.8 [315.2–490.2]	401.9 [302.8–499.7]	0.997
ACE2 (pg/mL)	1.30 [0.60–3.10]	1.53 [0.74–3.75]	2.1 [1.15–4.01]	2.42 [1.38–5.91]	0.059

*Note:* Values are presented as median [interquartile range]. *p*‐values represent age‐ and sex‐adjusted comparisons between healthy controls and post‐COVID‐19 patients at baseline using ANCOVA. IL‐12 was measured only in healthy controls.

Abbreviations: ACE2, angiotensin‐converting enzyme 2; IFN, interferon; IL, interleukin; IP‐10, interferon γ‐induced protein 10; TNF, tumor necrosis factor.

## 4 Discussion

The sustained elevation of IL‐1β observed in the present study suggests low‐grade immune activation in post‐COVID‐19 patients. While SARS‐CoV‐2 infection has been associated with inflammasome engagement [5], the modest absolute IL‐1β concentrations observed here are substantially lower than those typically seen in robust systemic NLRP3 activation. Importantly, circulating IL‐1β is an indirect marker that may not fully reflect tissue‐level inflammasome engagement [9], and thus our findings should not be interpreted as direct evidence of persistent inflammasome activation. Instead, our data are compatible with several mechanisms—including low‐grade systemic inflammation, residual immune activation with trained innate immunity [10], or partial/tissue‐restricted inflammasome engagement—though we cannot definitively distinguish among these possibilities with the current data. Future studies incorporating direct indices of inflammasome activity, such as IL‐18 [11], caspase‐1 activity, or ASC specks [12], will be required to clarify the specific molecular pathways involved.

Regardless of the specific pathway, the inflammatory response appears to be particularly relevant to cognitive and affective symptoms through specific mechanisms: IL‐1β impairs hippocampal function by decreasing neurogenesis and causing synapse loss, particularly in the dentate gyrus and CA1 regions, compromising both pre‐ and postsynaptic termini [13].

The age‐specific pattern of IL‐1β elevation in our cohort (20–59 years) provides insight into post‐COVID‐19 inflammatory responses. Whereas IL‐1β elevation occurs in various inflammatory conditions, SARS‐CoV‐2 infection may distinctly affect immune responses through chronic activation of inflammatory pathways [14]. Mitochondrial dysfunction has been proposed as a link between cellular stress and sustained inflammation in post‐COVID‐19 [4, 14]. Our findings of persistent IL‐1β elevation in younger individuals underscore the need for further research to clarify the underlying mechanisms.

Interpretation of IL‐1β levels relative to clinical benchmarks provides important context. Establishing universal reference ranges for IL‐1β remains challenging due to inter‐assay variability [15], and healthy individuals typically show undetectable or very low levels near analytical detection limits [16, 17]. Our HC showed a median IL‐1β of 0.24 pg/mL [IQR, 0.21–0.27], whereas post‐COVID‐19 patients demonstrated 0.50 pg/mL [IQR, 0.33–0.89]. These concentrations are substantially lower than acute severe COVID‐19 (hospitalized: 13.7 ± 5.8 pg/mL; intensive care unit patients: 40.8 ± 10.4 pg/mL) [18] or chronic inflammatory conditions such as rheumatoid arthritis (21.25 ± 4.19 pg/mL) [19]. However, the sustained elevation versus controls over 9 months without normalization supports biologically meaningful low‐grade inflammatory activity. This interpretation aligns with reports that IL‐1β may remain elevated up to 2 years in symptomatic individuals [20].

These findings from the PSCORE‐J provide evidence of sustained IL‐1β elevation in post‐COVID‐19 patients. While circulating IL‐1β is an indirect marker that cannot definitively identify specific inflammatory pathways, the consistent elevation may reflect ongoing immune dysregulation contributing to persistent neuropsychiatric symptoms.

## Author Contributions

Naoki Takamatsu and Hiroki Kimura contributed equally to this work as co‐first authors. Both led the study conceptualization, methodology development, and manuscript preparation. Naoki Takamatsu contributed to data analysis, while Hiroki Kimura made key contributions to data interpretation and critical discussions. Mari Oba provided expertise in statistical analysis, data visualization, and was instrumental in critical discussions. Hiroyuki Chiba, Ikue Umemoto, Yuki Moriyama, Nobuaki Matsunaga, Shinichiro Morioka, Daisuke Mori, Kazuhiro Hara, Aya Ogura, Kazufumi Yoshida, Hirohisa Watanabe, and Satoshi Maesawa contributed to data acquisition and patient recruitment. Masashi Ikeda, Masahisa Katsuno, Norio Ohmagari, Masaki Takao, Shinsuke Kito, Norio Ozaki, and Hironori Kuga provided senior oversight, including supervision, project administration, and funding acquisition. The manuscript was drafted collaboratively by Naoki Takamatsu and Hiroki Kimura, then critically reviewed and revised by all authors. All authors made substantial contributions to the work through data acquisition, analysis, or interpretation; participated in manuscript revision; provided final approval of the submitted version; and agreed to be accountable for all aspects of the work.

## Funding

This work was supported by the Japan Agency for Medical Research and Development (22dk0307115h0001, JP23gm1910005) and the intramural research grant for neurological and psychiatric disorders (5‐PSCORE, 3‐8, 6‐8).

## Ethics Statement

This study was approved by the Ethics Committee of the National Center of Neurology and Psychiatry (B2022‐052).

## Consent

Written or electronic informed consent was obtained from all participants.

## Conflicts of Interest

Shinsuke Kito has no conflicts of interest directly related to this study. However, over the past 3 years, Shinsuke Kito has received speaker's honoraria from Eisai Co. Ltd.; Inter Reha Co. Ltd.; Kowa Company Ltd.; Lundbeck Japan K.K.; Sumitomo Pharma Co. Ltd.; Otsuka Pharmaceutical Co. Ltd.; Takeda Pharmaceutical Co. Ltd.; Teijin Pharma Ltd.; and Viatris Inc.; consultant fees from Kyowa Pharmaceutical Industry Co. Ltd.; Teijin Pharma Ltd.; and Inter Reha Co. Ltd.; and research grants from Teijin Pharma Ltd. Norio Ozaki has received research support or speakers' honoraria from, or has served as a joint researcher with, or a consultant to, Sumitomo Pharma, Otsuka, Viatris, Eisai, Mochida, Kyowa Pharmaceutical Industry, Nihon Medi‐Physics, Nippon Chemiphar, Medical Review, Nippon Boehringer Ingelheim, SUSMED, outside the submitted work. Hirohisa Watanabe received lecture fees from Kyowa Kirin, Takeda Pharmaceutical, Sumitomo Pharma, Eisai, Ono Pharmaceutical, and AbbVie. All other authors declare no conflicts of interest.

## Data Availability

The data that support the findings of this study are not publicly available due to ethical restrictions and privacy concerns, as the research protocol and informed consent process did not include provisions for public data sharing. Furthermore, the data contain potentially sensitive information from psychiatric patients with long‐term follow‐ups. Additionally, these data were collected as part of the PSCORE‐J registry at the National Center of Neurology and Psychiatry. The data may be available from the corresponding author upon reasonable request and with approval from the Ethics Committee of the National Center of Neurology and Psychiatry.
